# Development of an algorithm for phenotypic screening of carbapenemase-producing Enterobacteriaceae in the routine laboratory

**DOI:** 10.1186/s12879-016-2174-y

**Published:** 2017-01-17

**Authors:** Jérôme Robert, Alix Pantel, Audrey Merens, Elodie Meiller, Jean-Philippe Lavigne, Marie-Hélène Nicolas-Chanoine, on behalf of ONERBA’s carbapenem resistance study group

**Affiliations:** 1 Sorbonne Universités, UPMC Univ Paris 06, INSERM, U1135, Centre d’Immunologie et des Maladies Infectieuses (CIMI), Eq 13, F-75013 Paris, France; 2 Bactériologie-Hygiène, Hôpitaux Universitaires Pitié Salpêtrière - Charles Foix, APHP, F-75013 Paris, France; 3 INSERM U1047, UFR de Médecine, Université Montpellier 1, Nîmes, France; 4 Service de Microbiologie, CHU Carémeau, Nîmes, France; 5 Laboratoire de biologie, Hôpital d’Instruction des Armées Bégin, Saint- Mandé, France; 6 Service de Microbiologie, Hôpital Beaujon AP-HP, Clichy, France; 7 Faculté de Médecine D. Diderot - Paris 7, Paris, France; 8 INSERM UMR 1137, Université Paris 7, Paris, France

**Keywords:** Disk diffusion method, Algorithm, Screening, Carbapenemase, Enterobacteriaceae

## Abstract

**Background:**

Carbapenemase-producing Enterobacteriaceae (CPE) are difficult to identify among carbapenem non-susceptible Enterobacteriaceae (NSE). We designed phenotypic strategies giving priority to high sensitivity for screening putative CPE before further testing.

**Methods:**

Presence of carbapenemase-encoding genes in ertapenem NSE (MIC > 0.5 mg/l) consecutively isolated in 80 French laboratories between November 2011 and April 2012 was determined by the Check-MDR-CT103 array method. Using the Mueller-Hinton (MH) disk diffusion method, clinical diameter breakpoints of carbapenems other than ertapenem, piperazicillin+tazobactam, ticarcillin+clavulanate and cefepime as well as diameter cut-offs for these antibiotics and temocillin were evaluated alone or combined to determine their performances (sensitivity, specificity, positive and negative likelihood ratios) for identifying putative CPE among these ertapenem-NSE isolates. To increase the screening specificity, these antibiotics were also tested on cloxacillin-containing MH when carbapenem NSE isolates belonged to species producing chromosomal cephalosporinase (AmpC) but *Escherichia coli*.

**Results:**

Out of the 349 ertapenem NSE, 52 (14.9%) were CPE, including 39 producing OXA-48 group carbapenemase, eight KPC and five MBL. A screening strategy based on the following diameter cut offs, ticarcillin+clavulanate <15 mm, temocillin <15 mm, meropenem or imipenem <22 mm, and cefepime <26 mm, showed 100% sensitivity and 68.1% specificity with the better likelihood ratios combination. The specificity increased when a diameter cut-off <32 mm for imipenem (76.1%) or meropenem (78.8%) further tested on cloxacillin-containing MH was added to the previous strategy for AmpC-producing isolates.

**Conclusion:**

The proposed strategies that allowed for increasing the likelihood of CPE among ertapenem-NSE isolates should be considered as a surrogate for carbapenemase production before further CPE confirmatory testing.

**Electronic supplementary material:**

The online version of this article (doi:10.1186/s12879-016-2174-y) contains supplementary material, which is available to authorized users.

## Background

Preventing the spread of carbapenemase-producing Enterobacteriaceae (CPE) is a priority because it may lead to therapeutic dead end [1]. The control strategies are based on better antibiotic use and decrease in cross-transmission. Thus, rapid detection of CPE in the laboratory is of paramount importance [2]. However, this detection is complex because carbapenemases display various hydrolytic activities with regard to β-lactams and mechanisms other than carbapenemase production, namely overproduction of AmpC β-lactamase and/or production of ESBL in isolates displaying reduced outer membrane permeability, are involved in resistance to carbapenems. In addition, appropriate studies have shown that the incidence of CPE is very low in some countries and that the other resistance mechanisms are the most frequent in carbapenem non-susceptible Enterobacteriaceae (NSE) [3–5]. Therefore, looking for CPE among all carbapenem NS isolates is difficult, and the routine clinical laboratory is in need of simple tools allowing for the rapid suspicion of CPE among carbapenem NSE isolates before further testing by more specific tests.

Recent tests designed to specifically detect carbapenemase production by real-time PCR assays [6] or carbapenem hydrolysis [7, 8] have been developed [9]. Nevertheless, these tests are not yet available in any microbiological laboratory or some may be costly if applied on all carbapenem NSE in a context of low CPE incidence. Thus, the objective is to decrease the number of carbapenem NSE submitted to such specific tests. For this purpose, a few teams sought to delineate laboratory screening strategies based on simple and widely available tests to identify isolates with high likelihood of being CPE among carbapenem NSE [10–15]. However, the effectiveness of these screening strategies has mostly been tested against biased samples, i.e. samples among which CPE were by far over-represented as compared to the current CPE prevalence [11, 12].

As we reported a low proportion (circa 10%) of CPE among non-selected carbapenem NSE in France in 2011–2012 [5], the French committee on antibiotic susceptibility testing (CA-SFM) affiliated to the European committee on antimicrobial susceptibility testing (EUCAST) raised interest in using this unbiased isolates collection to develop a screening strategy that could be applied in all laboratories to eliminate with certainty carbapenemase-negative isolates among carbapenem NSE. The aim of this article is to present the process used to establish a comprehensive screening algorithm, which was adopted by the French CA-SFM in 2015 (http://www.sfm-microbiologie.org/UserFiles/files/casfm/CASFM_EUCAST_V1_2015.pdf).

## Methods

### Bacteria

A total of 80 laboratories throughout the French territory participated on a voluntary basis in the study during a 6-month period in 2011–2012. As referral centre, we received 744 non-duplicate ertapenem NSE (ertapenem MIC >0.5 mg/L: broth microdilution method according to EUCAST recommendations) [16] consecutively collected, including 392 (52.7%) *Enterobacter cloacae*. Because of the very low prevalence of carbapenemase in *E. cloacae* at the period of the study [5], we randomly selected one fifth of the isolates belonging to this species for comprehensive analysis by using a roster ordered by laboratory and by isolation date. Therefore, 349 non-duplicate ertapenem NSE isolates, including 222 clinical isolates previously reported [5] and 127 consecutive isolates obtained from screening rectal swabs were studied. Isolates were characterised (species identification, antibiotic susceptibility and ESBL production) in each laboratory and further analysed by the referral centre, notably with regard to their *bla* gene content (Check-MDR CT103 array, Check-Points, Wageningen, The Netherlands) [5].

### Antibiotic susceptibility

The following antibiotic were tested by using the agar diffusion method following the 2011 CA-SFM recommendations on Mueller-Hinton agar medium (MH, Bio-Rad, Marne La Coquette, France) and on MH containing 250 mg/L cloxacillin (MH_cloxa_, bioMérieux, Marcy l’Etoile, France): ertapenem 10 μg, imipenem 10 μg, meropenem 10 μg, doripenem 10 μg, ticarcillin/clavulanate 75/10 μg, piperacillin/tazobactam 75/10 μg, cefotaxime 30 μg, ceftazidime 30 μg, cefepime 30 μg, and temocillin 30 μg [17].

Imipenem and meropenem MICs were determined by using the broth microdilution method in 96 wells-plaques containing lyophilized antibiotics (Sensititre MIC plates, Biocentric, Bandol, France).

### Additional phenotypic test designed for carbapenemase detection

Because previously described screening strategies [13, 15] included the use of the Rosco Diagnostica Neo-Sensitabs KPC and MBL confirm kit (Eurobio, Les Ullis France), each isolate of our collection was also submitted to this test according to manufacturer’s instructions. It consists of four tablets: tablet A containing meropenem, tablet B containing meropenem and dipicolinic acid [metallo-β-lactamase (MBL) inhibitor], tablet C containing meropenem and cloxacillin (AmpC inhibitor), and tablet D containing meropenem and boronic acid (class A carbapenemase KPC inhibitor). The inhibition zone diameter around tablet A was compared to that of each of meropenem-plus-inhibitor tablets (B, C, and D). If diameter around tablet B showed a difference >4 mm from that around tablet A, the isolate was recorded as demonstrating MBL activity. If diameter around tablet D showed a difference >4 mm from that of tablet A, the isolate was recorded as demonstrating KPC activity. If diameters around tablets C and D both showed a difference >4 mm from that of tablet A, the isolate was recorded as demonstrating AmpC activity coupled with outer membrane impermeability.

### Data analysis

The main objective of the analysis was to determine strategies based on the disk diffusion method allowing for retaining all carbapenemase-producing isolates (100% sensitivity, Se) and reducing the number of isolates submitted to CPE confirmatory tests, i.e. with the highest specificity. The performances, i.e. Se, specificity (Sp), positive (+) and negative (−) likelihood ratios (LR), were computed for single or combined screening tests with regard to their ability to separate carbapenemase-producing isolates from those non-producing carbapenemase.

These performances were successively evaluated with regard to clinical breakpoints delineating NS isolates from the susceptible (CA-SFM) ones, diameter cut-offs and specific carbapenemase detection test (Rosco test) previously published in strategies designed for screening of carbapenemase-positive isolates [13–15, 18], as well as diameter cut-offs determined in the present study by analysing the distributions of inhibition zone diameters displayed by our non-selected isolate collection (Additional file 1: Figure S1).

We used LR because they are independent of the pre-test probability of the event in the tested population (prevalence rate), in contrast with positive and negative predictive values. Moreover, by using the Fagan’s nomogram, each laboratory can derive the positive and negative predictive values of any test by using the LR of the test and the prevalence rate of the event before the test [19]. The LR of a positive test (LR+) is the ratio of the true-positive rate to the false-positive rate, i.e. Se/(1-Sp). The LR of a negative test (LR-) is the ratio of the false-negative rate to the true-negative rate, i.e. (1-Se)/Sp. Hence, the values of the LR range from zero to infinity. The farther the LR value is away from 1 (reference where the test has no diagnostic value), the stronger the interest of the test.

LR graphs derived from standard receiver operating characteristics curves analysis were used to visually compare the interest of “screening tests” (II) to a reference test (I) [20, 21]. Non-susceptibility to imipenem as defined by CA-SFM clinical breakpoint (imipenem inhibition diameter size <24 mm) was chosen as reference test because it is a widely used test in routine susceptibility testing. In LR graphs, the false-positive rate (1-Sp) of the reference (I) is plotted against its true-positive rate (Se). The slope of a solid line connecting this defined point to the point (0,0) represents the LR of a positive test, and the slope of a dashed line connecting this point to the point (1,1) represents the LR of a negative test (Fig. 2). Consequently, four regions are defined: region A, where the value of the screening test (II) yields overall superior characteristics than the reference (LR+ test II > LR+ test I and LR- test II < LR- test I); region B, where the value of the screening test (II) is superior to test I to eliminate the production of carbapenemase (LR+ test II < LR+ test I and LR- test II < LR- test I); region C, where the value of test II is superior for retaining isolates likely to produce of carbapenemase (LR+ test II > LR+ test I and LR- test II > LR- test I); region D, where the value of test II is inferior overall (LR+ test II < LR+ test I and LR- test II > LR- test I).

### Statistical analysis

The McNemar’s test with exact significance probability was used to compare Se and Sp of selected tests and strategies.

## Results

The characteristics of the 349 ertapenem-NS isolates are shown in Table 1. A total of 52 (14.9%) isolates harboured carbapenemase genes, and 179 (51.3%) produced ESBL. Carbapenemase-encoding genes were the most frequent in *Klebsiella pneumoniae* (27.3%) and *E. coli* (23.8%), while none of the *Enterobacter* spp harboured carbapenemase-encoding genes. Among the 52 carbapenemase-encoding genes, 39 (75.0%) encoded for the OXA-48 group carbapenemase, eight (15.4%) for KPC, and five (9.6%) for MBL. Imipenem MIC for the 52 CPE ranged from 1 mg/L to 64 mg/L, with 18 (34.6%) isolates displaying an imipenem MIC ≤ 2 mg/L. Meropenem MIC for CPE ranged from 0.25 mg/L to 64 mg/L, with 28 (53.8%) isolates displaying a meropenem MIC ≤ 2 mg/L. All CPE isolates with imipenem or meropenem MIC ≤ 2 mg/L produced OXA-48 group carbapenemases.

**Table 1:** Number and proportion of ESBL-producing and carbapenemase-producing Enterobacteriaceae included in the study according to species

Species	Number (%) of isolates					
	Total	ESBL+	Carbapenemase positive			
			Total	OXA-48	KPC	MBL
*Klebsiella pneumoniae*	121	91 (75.2)	33 (27.3)	24	7	2
*Enterobacter cloacae*	89	32 (36.0)	0			
*Enterobacter aerogenes*	46	20 (43.5)	0			
*Escherichia coli*	42	24 (57.1)	10 (23.8)	9		1
*Citrobacter freundii*	18	7 (38.9)	2 (11.1)	1	1	
*Serratia marcescens*	10	0	2 (20.0)	2		
*Klebsiella oxytoca*	5	0	2 (40.0)	2		
*Proteus mirabilis*	1	1 (100)	1 (100)			1
*Salmonella* spp	1	1 (100)	1 (100)			1
Others	16	3 (18.8)	1 (6.3)	1		
Total	349	179 (51.3)	52 (14.9)	39	8	5

*ESBL*+ extended-spectrum β-lactamase production, *OXA-48* OXA-48 group carbapenemase, *MBL* metallo-beta-lactamase

### Screening with a single antibiotic disk

When using the inhibition zone diameter of a single antibiotic disk and CA-SFM clinical breakpoints, non-susceptibility to carbapenems other than ertapenem yielded Se ranging from 32.7 to 42.3% (Table 2) to screen CPE among ertapenem NS isolates. Higher Se (98.1%) was obtained with piperacillin/tazobactam or ticarcillin/clavulanate clinical breakpoints but with very low Sp (25.9 and 1.7%, respectively) (Table 2). The use of cut-offs derived from a previous publication [14] for piperacillin/tazobactam (<16 mm) or from analysis of the inhibition zone diameter distribution in the present study (Additional file 1: Figure S1) for ticarcillin/clavulanate (<15 mm) allows increasing significantly Sp, although Sp values remained rather low (<65%). With regard to temocillin, a high Se, up to 96.2%, was reached when the previously proposed 12 mm inhibition zone diameter cut-off [14] or the 15 mm cut-off derived from our data analysis (Additional file 1: Figure S1) were applied (Table 2). The use of the previously proposed 8 mm temocillin cut-off [13] decreased the Se to 67.3% (Table 2).

**Table 2:** Sensitivity (Se, %), specificity (Sp, %), positive and negative likelihood ratios (LR+ and LR-, respectively) of screening strategies for carbapenemase-positive isolates among 349 isolates non-susceptible to ertapenem

Antibiotic and diameter (< in mm)	Number (%) of isolates		Se %	Sp %	LR+	LR-
	Carbase+ (*n* = 52)	Carbase- (*n* = 297)				
Clinical breakpoints						
IMI 24	22 (42.3)	44 (14.8)	42.3	85.2	2.84	0.68
MER 22	22 (42.3)	40 (13.5)	42.3	86.5	3.12	0.67
DOR 24	17 (32.7)	22 (7.4)	32.7	92.6	4.37	0.72
TAZ 21	51 (98.1)	220 (74.1)	98.1	25.9	1.31	0.11
TCC 24	51 (98.1)	292 (98.3)	98.1	1.7	0.99	1.53
FEP 24	42 (80.8)	138 (46.5)	80.8	53.5	1.73	0.37
Cut-offs						
MER 27 ^a^	40 (76.9)	80 (27.0)	76.9	73.0	2.83	0.32
TEM 8 ^a^	35 (67.3)	12 (4.0)	67.3	96.0	15.97	0.34
TAZ 16 ^b^	46 (88.5)	105 (35.4)	88.5	64.7	2.48	0.19
TEM 12 ^b^	43 (82.7)	65 (21.9)	82.7	78.1	3.73	0.23
MER 25 ^c^	32 (61.5)	59 (19.9)	61.5	80.1	3.07	0.48
TCC 15 ^d^	51 (98.1)	227 (76.4)	98.1	23.6	1.27	0.12
TEM 15 ^d^	50 (96.2)	126 (42.4)	96.2	57.6	2.24	0.08
FEP 26 ^d^	43 (82.7)	162 (54.6)	82.7	45.4	1.51	0.39
Combination of cut-offs						
TAZ 16 & TEM 12 ^b*^	39 (75.0)	48 (16.2)	75.0	58.9	4.62	0.08
TCC 15 & TEM 15 ^d^	50 (96.2)	94 (31.7)	96.2	68.3	3.00	0.07
TCC 15 & TEM 15 & IMI 22 ^d^	52 (100)	109 (36.7)	100	63.3	2.70	0.01
TCC 15 & TEM 15 & MER 22 ^d^	52 (100)	110 (37.0)	100	63.0	2.67	0.01
TCC 15 & TEM 15 & IMI 22 & FEP 26 ^d^	52 (100)	92 (31.0)	100	69.0	3.19	0.01
TCC 15 & TEM 15 & MER 22 & FEP 26 ^d^	52 (100)	93 (31.3)	100	68.7	3.16	0.01
Combination of cut-offs + additional tests						
TCC 15 & TEM 15 & IMI 22 & IMI 32-Clo ^d,e^	51 (98.1)	81 (27.3)	98.1	72.7	3.55	0.04
TCC 15 & TEM 15 & MER 22 & MER 32-Clo ^d,e^	52 (100)	68 (22.9)	100	77.1	4.31	0.01
TCC 15 & TEM 15 & IMI 22 & FEP 26 ^d^ & IMI 32-Clo ^d,e^	51 (98.1)	71 (23.9)	98.1	76.1	4.05	0.04
TCC 15 & TEM 15 & MER 22 & FEP 26^d^ & MER 32-Clo^d,e^	52 (100)	63 (21.2)	100	78.8	4.65	0.01
MER 25 & TEM 11 & Rosco confirm kit ^c,f^	29 (55.8)	23 (9.4)	55.8	90.6	5.82	0.49
MER 27 & TEM 8 & Rosco confirm kit ^a,g^	36 (69.2)	14 (4.7)	69.2	95.3	14.15	0.33

*IMI* imipenem, *MER* meropenem, *DOR* doripenem, *FEP* cefepime, *TAZ* piperacillin/tazobactam, *TCC* ticarcillin/clavulanate, *TEM* temocillin, *Carbase*+ or *Carbase*- carbapenemase-positive or -negative isolate

^a^reference [13]; ^b*^85 (24.4%) isolates not classified according to reference [14] strategy; ^c^reference [15]; ^d^this study

^e^cloxacillin test applied only to chromosomally encoding AmpC Enterobacteriaceae

^f^258 (73.9) of our isolates not tested according to reference [15] because of meropenem diameter ≥25 mm towards these isolates

^g^ 229 (65.6%) of our isolates not tested according to reference [13] because of meropenem diameter ≥27 mm towards these isolates

### Screening with a combination of antibiotic disks

The previously published combination [14] of inhibition zone diameter of piperacillin/tazobactam <16 mm and temocillin <12 mm yielded a Se of 75.0% but 24.4% of our isolates were not classified because they did not fit in any of the categories defined by the authors (Table 2). Combining ticarcillin/clavulanate <15 mm with temocillin <15 mm as derived from the analysis of zone diameters for our isolates collection increased Se (96.2%) and Sp (68.3%). Se of 100% and Sp of 63% were reached when considering an inhibition zone diameter of imipenem or meropenem <22 mm for isolates not retained by ticarcillin/clavulanate <15 mm and temocillin <15 mm (Table 2). Finally, the Sp of the latter strategy was significantly improved (69 vs 63%, *p* < 0.001; Table 3) by retaining only isolates with cefepime inhibition zone diameter <26 mm for the species producing chromosomal AmpC but *E. coli* (Table 2). All isolates were classified by using our strategies.

**Table 3:**
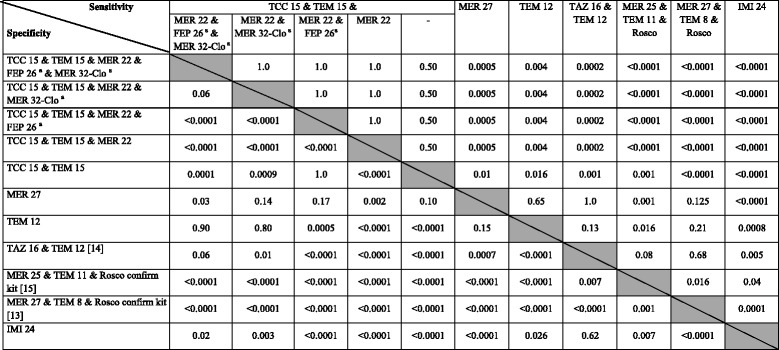
*P*-values (McNemar’s test) comparing sensitivities and specificities of selected strategies for the 349 ertapenem non-susceptible Enterobacteriaceae isolates

*TCC* ticarcillin/clavulanate, *TEM* temocillin, *MER* meropenem, *FEP* cefepime, *TAZ* piperacillin/tazobactam, *IMI* imipenem, *Clo* Mueller-Hinton agar containing cloxacillin

^a^test applied only to Enterobacteriaceae chromosomally encoding Amp-C

### Screening with the classical disc diffusion method and additional tests

**Fig. 1:**
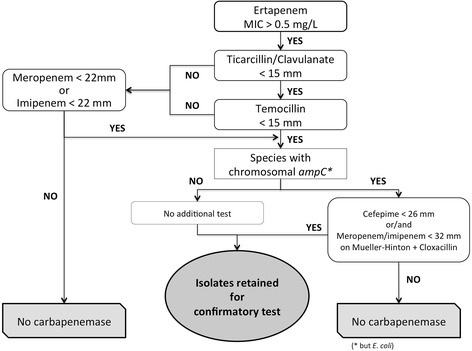
Proposed strategy to screen carbapenemase-producing Enterobacteriaceae among ertapenem non-susceptible isolates

When analysing the results of the different tests obtained on our collection of isolates, the best combination allowing for reaching a Se of 100% and Sp of 78.8% consisted of the combination of the disk diffusion method (temocillin <15 mm, ticarcillin/clavulanate <15 mm and, imipenem or meropenem <22 mm, cefepime <26 mm), and an additional screening, namely meropenem or imipenem <32 mm on MH containing cloxacillin (Table 2 and Fig. 1) for the species producing chromosomal AmpC but *E. coli*.

The strategy proposed by van Dijk et al. [15], which combines temocillin <11 mm and the Rosco confirmatory test with regard to isolates selected on meropenem MIC ≥0.5 mg/L (i.e. estimated inhibition zone diameter <25 mm) [14], displayed a Se of 55.8% and a Sp of 90.6% in our collection of isolates (Table 2). It has to be noted that 73.9% of the isolates of our study, including 20 of the 52 CPE, would not have been tested because they displayed a meropenem inhibition zone diameter ≥25 mm, which is used as first screening criteria by the authors (Additional file 1: Figure S1).

The strategy proposed by Hrabak et al. [13], which combines temocillin <8 mm and the Rosco confirmatory test with regard to isolates selected on meropenem MIC ≥0.25 mg/L (i.e. estimated inhibition zone diameter <27 mm) [14] displayed a Se of 69.2% and a Sp of 95.3% in our collection of isolates. However, 65.6% of our isolates, including 12 of the 52 CPE, would not have been tested because they displayed a meropenem inhibition zone diameter ≥27 mm, which is used as first screening criteria by the authors (Additional file 1: Figure S1).

### Comparison of screening tests or strategies by using a likelihood-ratios graph

**Fig. 2:**
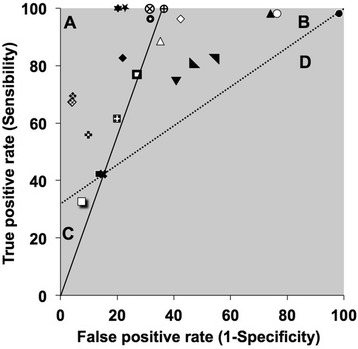
Comparison of characteristics of single or combined tests compared to imipenem non-susceptible clinical category (**x**, imipenem 24) by using likelihood ratio graphs: ● Ticarcillin/clavulanate 24; ○ Ticarcillin/clavulanate 15; ▲ Piperacillin/tazobactam 21; ∆ Piperacillin/tazobactam 16; ■ Meropenem 22; ▣ Meropenem 25;  Meropenem 27; ❏ Doripenem; ◈ Temocillin 8; ♦ Temocillin 12; ◊ Temocillin 15; ◣ Cefepime 24; ◥ Cefepime 26; ▼ Temocillin 12 & Piperacillin/tazobactam 16 [14];  Temocillin 15 & Ticarcillin/clavulanate 15; ⨁ Temocillin 15 & Ticarcillin/clavulanate 15 & Imipenem 22/Temocillin 15 & Ticarcillin/clavulanate 15 & Meropenem 22; ⨂ Temocillin 15 & Ticarcillin/clavulanate 15 & Imipenem 22 and Cefepime 26 for AmpC producers/Temocillin 15 & Ticarcillin/clavulanate 15 & Meropenem 22 and Cefepime 26 for AmpC producers;  Temocillin 11 & Rosco KPC-MBL confirm kit [15]; ❖ Temocillin 8 & Rosco KPC-MBL confirm kit [13]; ★□ Temocillin 15 & Ticarcillin/clavulanate 15 & Meropenem 22 & Meropenem 32 on Mueller-Hinton-cloxacillin for AmpC producers; ✶ Temocillin 15 & Ticarcillin/clavulanate 15 & Meropenem 22 and Cefepime 26 & Meropenem 32 on Mueller-Hinton-cloxacillin for AmpC producers

The overall performances of selected strategies were plotted on a LR graph (Fig. 2) taking as reference non-susceptibility to imipenem (<24 mm) because it is a widely used test. Numerous strategies fell in the B zone, indicating that they work better than imipenem <24 mm for eliminating non-CPE isolates.

Doripenem <24 mm fell into the C zone, indicating that it performs better than the reference for retaining CPE.

All other strategies fell into the A zone indicating that they perform better than NS to imipenem alone for both selecting CPE and eliminating non-CPE isolates. Among all latters, the best characteristics (LR+ and LR-) were for the combinations temocillin <15 mm + ticarcillin/clavulanate <15 mm + meropenem or imipenem <22 mm + meropenem <32 mm on MH-cloxacillin, and temocillin <15 mm + ticarcillin/clavulanate <15 mm + meropenem or imipenem <22 mm and cefepime <26 mm + meropenem <32 mm on MH-cloxacillin. After full implementation, the two latter strategies retained a total of 120 (34.4%) or 115 (33.0%) putative CPE isolates for further carbapenemase confirmatory tests, respectively. Hence, the post-strategy CPE proportion reached 43.3 to 45.2% as compared to the 14.9% CPE pre-test proportion.

Better LR- (i.e. higher specificity) was reached for temocillin <12 mm + Rosco KPC-MBL confirm kit [15] and temocillin <8 mm + Rosco KPC-MBL confirm kit [13]. When applied on ertapenem NS isolates, these two strategies allowed for retaining less isolates (*n* = 50 and *n* = 52, respectively) for further testing than our strategies (*n* = 120 and *n* = 115) but missed from 16 (30.8%) to 23 (44.2%) of the 52 CPE.

Applying the most comprehensive strategy, i.e. temocillin <15 mm + ticarcillin/clavulanate <15 mm + meropenem or imipenem <22 mm and cefepime <26 mm + meropenem <32 mm on MH-cloxacillin to our population with an initial pre-test probability or CPE prevalence of 14.9%, a LR+ value of 4.65 (Table 2) will be translated into a positive post-test probability or positive predictive value (PPV) of 45% (Additional file 2: Table S1), and a LR- value of 0.01 into a negative post-test probability or negative predictive value (NPV) close to 100% (Additional file 2: Table S1).

### Inoculum size

After our study, EUCAST issued new guidelines for susceptibility testing, which recommends a larger inoculum size than the CA-SFM recommendations used for our study [16, 17]. This may question the validity of our strategies from now on. Hence, we compared inhibition zone diameters for temocillin and meropenem obtained by EUCAST and CA-SFM methods on all CPE and a sample of carbapenemase-negative isolates of our collection. The correlation between both methods was very high for both antibiotics and both groups of isolates (data not shown) and all CPE isolates were classified into the same groups by both methods when using the cut-offs of our strategies. Ticarcillin/clavulanate diameters were not assessed by using EUCAST recommendations, because all CPE isolates had diameters <15 mm by CA-SFM method, which implies that diameters will be similar or smaller with EUCAST larger inoculum size. In addition, Dortet et al. recently validated our algorithm by using the larger EUCAST inoculum and testing other carbapenemase-producing isolates [22].

## Discussion

Carbapenemase detection among carbapenem NSE is a major challenge in the routine laboratory. We designed screening strategies in order to retain 100% of carbapenemase-positive isolates while reaching the highest possible specificity by using the disc diffusion method. To reach our goal, we used data-driven cut-offs of diameter zones for commonly tested antibiotics including temocillin, and combined them to design an algorithm. Our strategies, which have been compared to those proposed by others using a similar approach [13–15], are shown to be highly relevant when sensitivity is the priority.

Our study and that of Huang et al. [14] are based on a collection of unselected isolates provided by a large panel of laboratories throughout the territory. Hence, reported performances are likely to be closed to those observed in the routine laboratory on the opposite to studies that used either very selected collections of isolates with sometimes undefined selection criteria or collections derived, for theoretical strategies, from heterogeneous publications and not tested in the routine laboratory [11, 13].

Our first strategy combining temocillin <15 mm + ticarcillin/clavulanate <15 mm + meropenem or imipenem <22 mm and cefepime <26 mm, was designed to be easily implemented in the routine laboratory and to screen isolates in a single step from the disc diffusion antibiogram at the same time as carbapenem non-susceptibility is identified. This strategy confirms the interest of temocillin combined to other tests in the screening of CPE [13–15]. However, we used a higher temocillin cut-off value, which was found to increase sensitivity. By using cefepime in this strategy, the specificity of the algorithm increased from 63 to 68.7%. Consequently, 58.5% of the total isolates were not retained for confirmatory tests as compared to 53.6% without cefepime. When validating our algorithm, the French reference centre for antibiotic resistance did not use cefepime [22]. Because of the large number of *Enterobacter* spp sent to this reference centre, it is likely that the use of cefepime would help decreasing the number of isolates submitted to a confirmatory test. To further increase the specificity of our algorithm, we used imipenem or meropenem disks on MH containing cloxacillin. This takes an additional day, similarly to strategies proposed by others who used assays designed to detect carbapenemase production [13, 15]. Of note, this additional test allowed for eliminating 67% of the total of carbapenem NSE. As a result, almost one half (45%) of retained isolates submitted to confirmatory tests are carbapenemase positive. By increasing the proportion of CPE among carbapenem NSE, the positive predictive value of confirmatory tests will significantly be increased.

Our study has some weaknesses. First, our collection of isolates does not contain the less frequent carbapenemases because isolates were prospectively and consecutively selected from routine laboratories. For instance, GES type enzymes are lacking, mainly because to date it has not been identified in France [23]. Nevertheless, the designed strategies we are likely to miss such isolates because ertapenem MIC towards GES-producing isolates are often lower than the chosen cut-off (MIC > 0.5 mg/L) [8, 24]. Similarly, for a few OXA-48-producing Enterobacteriaceae and notably *K. pneumoniae*, ertapenem MIC is ≤0.5 mg/L. Second, we chose to analyse a randomly selected sample of all carbapenem-NS *E. cloacae* collected during the study-period. Among those isolates, none was found to produce carbapenemase, and we were not able to challenge our screening strategies against carbapenemase-positive *E. cloacae*. However, the recent study of Dortet et al. proved that our algorithm applied in a reference centre did not miss any of the 19 carbapenemase-producing *Enterobacter* spp among the 214 *Enterobacter* spp isolates tested [22]. Third, the study was performed by voluntary laboratories, that, although rather numerous, may not be representative of all French laboratories. Finally, it should be noticed that since we performed our study, new or updated commercialized versions of carbapenem hydrolysis assays have been issued and should be further tested against our strategies [25, 26].

## Conclusions

In conclusion, our study proposes strategies to screen candidates for being CPE among a large panel of carbapenem-NS isolates. The advantages of these screening strategies are to reach 100% sensitivity by keeping a rather satisfactory specificity and to be applied in the routine laboratories directly from the disk diffusion method. The strategies should be considered as a tool for selecting isolates that will be further tested with more refined techniques such as PCR-based assays or assays based on the carbapenemase activity detection. For the latter, the advent of newer and cheaper versions of these assays should facilitate their use in most laboratories, mainly after screening. The proposed strategies will also help decreasing unnecessary implementation of isolation precautions in the hospital setting.

## Additional files

Additional file 1: Figure S1. Distribution of inhibition zone diameters for ticarcillin/clavulanate (A), piperacillin/tazobactam (B), cefepime (C), temocillin (D), imipenem (E), and meropenem (F). (JPG 87 kb)
Additional file 2: Table S1. Formulas to calculate post-test probabilities with positive and negative likelihood ratios (LR^+^ and LR^−^). (DOCX 11 kb)

## Abbreviations

CA-SFM: French committee on antibiotic susceptibility testing
CPE: Carbapenemase-producing Enterobacteriaceae
ESBL: Extended-spectrum beta-lactamase
EUCAST: European committee on antimicrobial susceptibility testing
LR: Likelihood ratio
MBL: Metallo-β-lactamase
MH: Mueller-Hinton agar medium
MH_cloxa_: Mueller-Hinton agar medium containing 250 mg/L cloxacillin
MIC: Minimal inhibitory concentration
NS: Non-susceptible
NSE: Non-susceptible Enterobacteriaceae
PCR: Polymerase chain reaction
Se: Sensitivity
Sp: Specificity
