# Supplementary figures and images for: Development of an algorithm for phenotypic screening of carbapenemase-producing Enterobacteriaceae in the routine laboratory
Source: BMC Infect Dis. 2017 Jan 17;17:78. doi: 10.1186/s12879-016-2174-y (PMC5240403; doi:10.1186/s12879-016-2174-y)

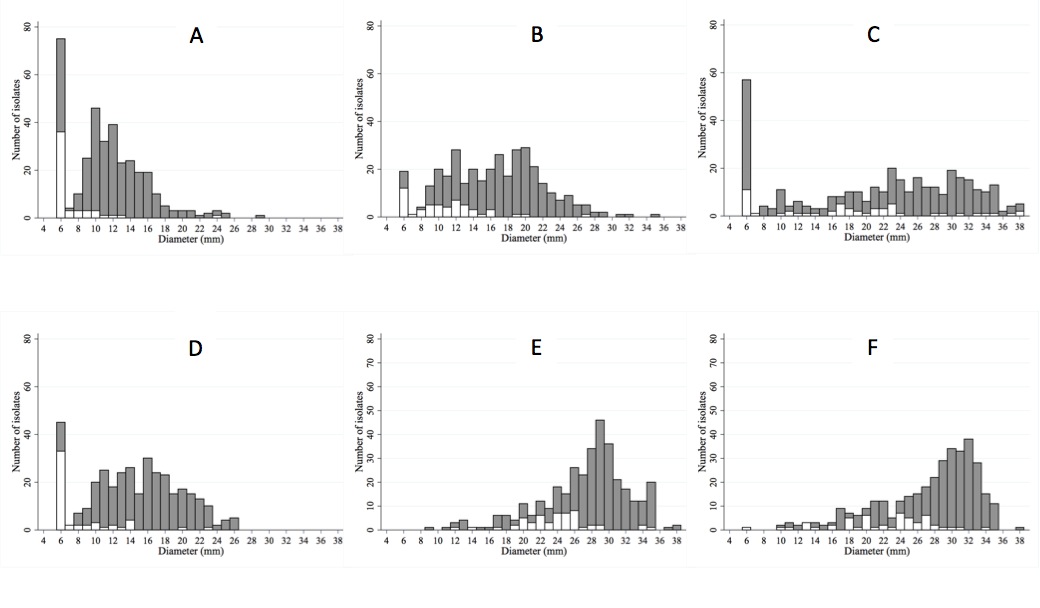

Supplement: Additional file 1: Figure S1. — Distribution of inhibition zone diameters for ticarcillin/clavulanate (A), piperacillin/tazobactam (B), cefepime (C), temocillin (D), imipenem (E), and meropenem (F). (JPG 87 kb) [file 12879_2016_2174_MOESM1_ESM.jpg]
