# Supplementary material for: Development of an algorithm for phenotypic screening of carbapenemase-producing Enterobacteriaceae in the routine laboratory
Source: BMC Infect Dis. 2017 Jan 17;17:78. doi: 10.1186/s12879-016-2174-y (PMC5240403; doi:10.1186/s12879-016-2174-y)
Supplement: Additional file 2: Table S1. — Formulas to calculate post-test probabilities with positive and negative likelihood ratios (LR+ and LR−). (DOCX 11 kb) [file 12879_2016_2174_MOESM2_ESM.docx]

**Table S1. Formulas to calculate post-test probabilities with positive and negative likelihood ratios (LR^+^ and LR^-^)**

PreTest probability = prevalence rate

PreTest odds = ${PreTest probabilty}/{(1-PreTest probability)}$

Positive PostTest odds = PostTest odds^+^ = $PreTest$ odds x LR^+^

Negative PostTest odds = PostTest odds^-^ =$PreTest$ odds x LR^-^

Positive PostTest Probability = ${{PostTest odds}^{+}}/{{1+PostTest odds}^{+}}$

Negative PostTest Probability = ${{PostTest odds}^{-}}/{{1+PostTest odds}^{-}}$
